
JOURNAL INFORMATION
==============================
NLM Title Abbreviation: ScientificWorldJournal
Journal ID: ScientificWorldJournal
NLM Journal ID: 101131163
Journal ID: TSWJ

The Scientific World Journal

ISSN: 2356-6140
EISSN: 1537-744X
Publisher: Wiley

ARTICLE INFORMATION
==============================
PMCID: PMC5763969
PMID: 20364242
DOI: 10.1100/tsw.2010.60
Article ID: 695239
Article version: 1
Subjects: Clinical Image

Lipodermatosclerosis: A Commonly Misdiagnosed Complication of Chronic Venous Insufficiency

Fallahzadeh Mohammad Kazem
Khalesi Mohammad
Namazi Mohammad Reza *
Medicinal and Natural Products Chemistry Research Center and Dermatology Department, Shiraz University of Medical Sciences, Shiraz, Iran
*Mohammad Reza Namazi: Namazi_mr@yahoo.comAcademic Editor: Daniel Batlle

Publication date: 2010
Electronic publication date: 2010 Apr 1
Volume: 10
First page: 576
Last page: 577
Received 2010 Feb 11; Revised 2010 Mar 12; Accepted 2010 Mar 15
Copyright: Copyright © 2010 Mohammad Kazem Fallahzadeh et al.
Copyright year: 2010
License: This is an open access article distributed under the Creative Commons Attribution License, which permits unrestricted use, distribution, and reproduction in any medium, provided the original work is properly cited.
License URL: https://creativecommons.org/licenses/by/3.0/

Keywords: lipodermatosclerosis, venous insufficiency, panniculitis, fibrosis

==============================
Clinical Image
*TheScientificWorldJOURNAL* (2010) 10, 576–577
ISSN 1537-744X; DOI 10.1100/tsw.2010.60

TheScientificWorldJOURNAL
www.thescientificworld.com

### Lipodermatosclerosis: A Commonly Misdiagnosed Complication of Chronic Venous Insufficiency

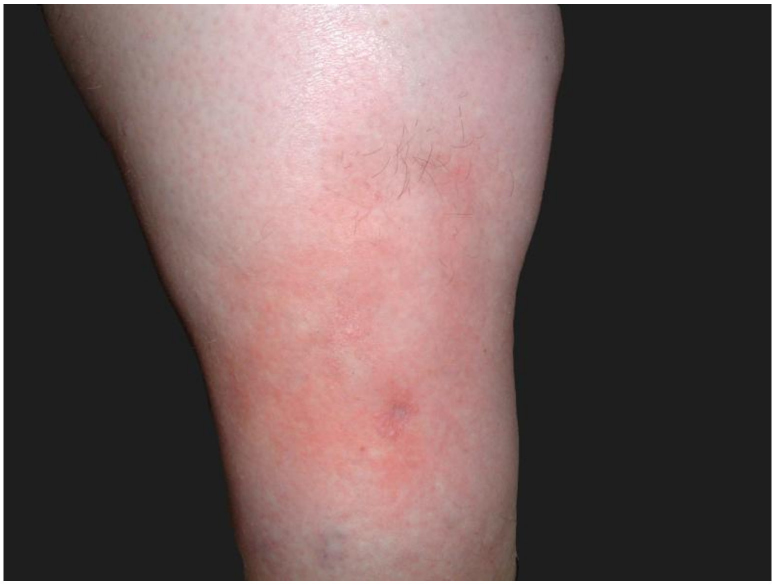

**Figure.** Lipodermatosclerosis on the left lower leg of a patient suffering from chronic venous insufficiency.

Mohammad Kazem Fallahzadeh, Mohammad Khalesi,
and Mohammad Reza Namazi*

*Medicinal and Natural Products Chemistry Research Center and Dermatology Department, Shiraz University of Medical Sciences, Shiraz, Iran*

**E-mail**: Namazi_mr@yahoo.com

*Received February 11, 2010; Revised March 12, 2010; Accepted March 15, 2010; Published April 1, 2010*

**KEYWORDS:** lipodermatosclerosis, venous insufficiency, panniculitis, fibrosis

A 50-year-old woman with a history of chronic venous disease presented with erythematous, painful, tense, bound-down plaques on the medial aspects of her lower legs, which were diagnosed as lipodermatosclerosis.

*Corresponding author.
©2010 with author.
Published by TheScientificWorld; www.thescientificworld.com

Lipodermatosclerosis is a complication of severe chronic venous insufficiency that results from high venous pressure and resulting increased capillary permeability, perivascular fibrin cuffing, and tissue hypoxia. These events culminate in fibrosis and membranous fat necrosis[1].

Lipodermatosclerosis can present as painful, red, indurated plaques that may be easily misdiagnosed as cellulitis, thrombophlebitis, and morphea[2]. It initially develops on the medial aspect of the ankle and then spreads to involve the entire leg circumferentially[3]. In its advanced states, lipodermatosclerosis, along with a lymphedematous upper portion of the leg and an edematous foot, can look like an inverted champagne bottle[3].

Compression therapy, drugs such as stanozolol, and surgical procedures are the current therapeutic options available for this recalcitrant conundrum[2].

### REFERENCES

1. Alguire, P.C. and Mathes, B.M. (2009) Pathophysiology of chronic venous disease. *Uptodate 17.3 Online*. Address: http://www.uptodate.com/online/content/topic.do?topicKey=vascular/5304&selectedTitle=1~7&source=search_result [accessed: February 10, 2010].
2. Heymann, W.R. (2009) Lipodermatosclerosis. *J. Am. Aca.d Dermatol*. **60,** 1022–1023.
3. Alguire, P.C. and Mathes, B.M. (2009) Epidemiology and clinical manifestations of chronic venous disease**.** *Uptodate 17.3 Online*. Address: http://www.uptodate.com/online/content/topic.do?topicKey=vascular/3010&selectedTitle=1~55&source=search_result [accessed: February 10, 2010].

**This article should be cited as follows:**

Fallahzadeh, M.K., Khalesi, M., and Namazi, M.R. (2010) Lipodermatosclerosis: a commonly misdiagnosed complication of chronic venous insufficiency. *TheScientificWorldJOURNAL* **10**, 576–577. DOI 10.1100/tsw.2010.60.
